# 电磁导航支气管镜在外周肺病变诊治中的临床应用进展

**DOI:** 10.3779/j.issn.1009-3419.2020.102.04

**Published:** 2020-06-20

**Authors:** 求名 陈, 舟 安, 钧 程, 望 吕, 坚 胡

**Affiliations:** 310003 杭州，浙江大学医学院附属第一医院胸外科 Department of Thoracic Surgery, the First Affiliated Hospital, Zhejiang University School of Medicine, Hangzhou, 310003, China

**Keywords:** 外周肺病变, 电磁导航支气管镜, 微创, 进展, Peripheral pulmonary lesions, Electromagnetic navigation bronchoscopy, Minimally invasive, Advances

## Abstract

随着计算机断层扫描的筛查应用普及，越来越多的外周肺部病变被发现，外周肺病变的诊断和治疗是目前临床较为棘手的问题之一。电磁导航支气管镜以电磁定位技术为基础，经支气管到达外周肺部病变进行活检或治疗，为肺部可疑病灶提供了新的微创诊治方法。本文将综述电磁导航支气管镜在外周肺病变诊治中的临床应用现状及进展。

## Abstract (en)

The incidence of peripheral pulmonary lesions (PPLs) is growing following the adoption of lung cancer screening by low-dose chest CT. The diagnosis and treatment of pulmonary nodules is one of the most difficult problems. Based on the electromagnetic positioning technology, the electromagnetic navigation bronchoscope is guided to the pulmonary nodules for biopsy or treatment, providing a new minimally invasive diagnosis and treatment method for suspicious lung lesions. This paper provides an overview of the current status and progress of electromagnetic navigation bronchoscopy in the diagnosis and treatment of peripheral pulmonary diseases.

随着影像技术的发展、计算机断层扫描（computed tomography, CT）的筛查应用普及，越来越多的外周肺部病变（peripheral pulmonary lesions, PPL）被发现，由于存在一定的恶性概率，PPL的诊断和治疗是目前临床较为棘手的问题之一。X线、CT、磁共振等无创检查对其诊断有一定参考意义，但无法明确诊断，支气管镜穿刺活检、支气管镜肺泡灌洗等有创检查的阳性率也不令人满意。而CT引导下经胸壁穿刺活检有较高诊断率，但气胸、出血等并发症发生率高，部分病例难以穿刺成功，且增加了患者辐射暴露^[1]^。电磁导航支气管镜（electromagnetic navigation bronchoscopy, ENB）的问世为解决此难题提供了良好方法，它是一种将电磁导航系统与现有高清支气管镜系统相结合的新技术，以电磁定位技术为基础，结合高分辨率螺旋CT成像与计算机虚拟支气管镜技术，经支气管镜引导至PPL进行活检或治疗，为肺部可疑病灶进行精确诊断，突破了传统支气管镜仅能进入段支气管的技术瓶颈，提供了新的微创诊治方法。本文将对ENB的历史发展、电磁导航技术原理及构成、临床应用现状及进展做一综述。

## 1 ENB的历史发展

Solomon等^[2]^1998年首次报道应用ENB进行动物实验研究，证实ENB实时定位有助于经支气管穿刺针吸活检获取肺外周支气管病变标本。2000年，Solomon等^[3]^首次报道在人体使用ENB检查，并在15例临床病例应用ENB技术定位，发现气管内定位法优于体表定位法。曾为以色列国防开发了电磁导航定位系统的Gilboa等1998年将该技术应用于心脏介入治疗领域，2001年应用于肺部介入治疗领域^[4]^。2002年Schwarz等^[5]^在以色列利用该系统开展了首例临床前研究，顺利探及全部人工病灶且无并发症，随后在2003年开展了首次临床研究，13例患者中9例活检结果阳性，并逐步证实了ENB为一种安全有效的检查技术，因而第一代ENB系统先后在欧洲（2002年）和美国（2004年）使用，2007年第3代ENB系统获得美国食品与药品监督管理局（Food and Drug Administration, FDA）批准。2013年国内医院获得ENB进口许可，逐渐开展ENB相关临床工作，目前已有数十家医院开展相关临床研究和工作，2014年复旦大学附属中山医院开展了国产ENB的临床研究^[6]^。

## 2 电磁导航支气管镜技术原理及构成

ENB的设计原理源自全球定位系统（global positioning system, GPS），是现代影像技术、计算机技术与立体定向技术有机结合的成果。在电磁导航出现之前，图像导航手术领域主要是利用机械和光学系统开发，可跟踪手术器械，主要应用于神经外科等领域（头部固定，不受呼吸及心跳影响）^[7]^。电磁跟踪定位系统包括磁场发生器和定位传感器：磁场发生器由线圈构成，在三维空间形成交变振荡的低频磁场；而定位传感器非常小，定位传感器在磁场中运动时，通过切割磁力线产生变化的电流，定位系统进而可根据电流大小和变化情况获知定位传感器的位置，与CT图像重建形成的三维虚拟支气管图像叠加调整，从而引导至目标病灶。

ENB主要包括：①电磁定位板，可产生低频均匀电磁场，置于检查床床垫下，使患者胸部处于电磁场中；②导航探头：导航探头固定于一段可弯曲导管的尖端，直径1 mm，长8 mm，可360°旋转，在电磁场中其方位如X、Y、Z轴及倾斜、转动等运动可被定位系统获取并传至计算机；③扩展操作通道：可置入相关操作器械由导航系统引导至靶区进行操作；④ENB系统主机与显示器：通过计算机硬件平台接收和处理磁导航信号、处理和显示支气管镜下的实际图像与虚拟导航图像，从而引导和观察探头的位置和走向。

ENB操作主要包括两部分：①术前路径规划：通过软件将CT原始图像数据进行三维重建产生虚拟支气管图像，找到目标病灶标记，选择5个-7个解剖标记，生成通往目标病灶的导航路径；②术中气管内导航：患者麻醉后，医生操作电子支气管镜，通过扩展通道置入定位导管，将虚拟气管镜图像上选定的标记与体内探头位置确认，虚拟图像与实际气管镜下图像匹配，根据术前的规划路径到达目标病灶，导航结束取出定位探头，经工作通道进行针吸、活检、刷检、注入染料定位、治疗等操作。

## 3 电磁导航支气管镜临床应用现状及进展

### 3.1 诊断

越来越多的临床研究证实，通过ENB可对PPL活检取样，对明确病理诊断具有重要价值。2006年Gildea等^[8]^发表了在美国开展的首次大规模前瞻性ENB临床研究，ENB引导下经支气管肺部病灶取样成功率为74%（40/54），恶性病变确诊率为74.4%（32/43）。Gex等^[9]^2014年荟萃分析15项临床研究（1, 033个肺部病变）评价ENB诊断肺部结节的准确率和安全性，发现64.9%结节可获得明确诊断，整体诊断准确率为73.9%；肺癌诊断的灵敏度为71.1%。而Zhang等^[10]^荟萃分析17项临床研究（1, 106例患者）显示ENB诊断敏感度和特异度分别是82%和100%。2013年Ha等^[11]^回顾性分析65例经ENB诊断为肺癌的患者资料，ENB取样活检与手术标本组织病理学一致性达87.5%，15例腺癌患者中的14例标本量足以进行表皮生长因子受体（epidermal growth factor receptor, *EGFR*）突变检测。NVIGATE研究^[12]^中86%的患者可获取足够标本用于基因检测。这些研究证实ENB可获取足够标本用于病理诊断和基因检测等。

目前已有数十项ENB相关研究^[4]^发表，其诊断率分布在33%-97%，大部分报道为67%-84%，波动范围较大，这与操作者经验、ENB的精准性、有无联合径向支气管内超声（endobronchial ultrasound, EBUS）、PPL位置和大小、有无支气管充气征等有关。Seijo等^[14]^发现存在支气管充气征患者的ENB诊断率明显高于无支气管充气征患者（79%*vs*31%）。Gex等^[9]^分析发现PPL位于肺上叶或中叶、存在支气管充气征、ENB系统虚拟与实际注册误差低、联合使用径向EBUS或鞘管吸引取样等可有助于改善诊断准确率。Eberhardt等^[14]^在2007年发表一项前瞻性随机对照研究结果，发现联合应用ENB和径向EBUS可提高诊断准确率（88%），明显高于单独使用ENB组（59%）和径向EBUS组（69%），分析原因为ENB可实时导航，而径向EBUS使病灶可视化，从而实时确认，两者结合促使ENB更精准有效。Karnak等^[15]^结合快速现场评价（rapid on-site evaluation）有助于提高ENB诊断准确率，Mukherjee等^[16]^发现使用一种尖端弯曲的改良导管有助于提高ENB诊断准确率。针对电磁导航精准程度受肺部呼吸运动影响Flenaugh等^[17]^分享了使用另一套ENB导航经验，该系统纳入吸气相/呼气相的CT图像，并追踪操作过程中的呼吸运动，确保操作过程中动态校准，降低呼吸对结节定位的干扰；升级活检工具也带电磁定位，确保整个过程实时跟踪，避免盲目取样。

既往研究多数存在一定局限性，如样本量小、选择偏差和回顾性设计等。迄今为止规模最大的前瞻性多中心ENB研究——NAVIGATE研究^[12]^于2018年发表，共纳入29家医院的1, 215例患者，49.1%的PPL直径小于20.0 mm。该研究最大的亮点是有严格的一年随访结果，确保阴性或不确定的结果为真阴性结果。该研究的ENB路径规划时间5 min，而ENB操作时间约25 min，94%的患者完成了ENB导航并成功获取标本，1个月和1年的随访完成率分别为99%和80%，1年后确认的诊断准确率为73%。ENB辅助获取的标本中44%为恶性，诊断恶性肿瘤的敏感性、特异性、阳性预测值和阴性预测值分别为69%、100%、100%和56%。无支气管充气征患者的诊断率亦可达67%，与近年的ENB系统精准性提高、操作者经验增加和取材工具性能升级有关。

ENB的优势是安全微创，经自然腔道——支气管到达PPL活检。Gex等^[9]^对15项临床研究的荟萃分析，气胸发生率为3.1%，需留置胸管的仅为1.6%。NAVIGATE研究^[12]^报道的总体气胸发生率为4.3%，但需住院或留置胸管的气胸仅为2.9%，2级以上出血和4级以上呼吸衰竭发生率分别为1.5%和0.7%。正因为ENB安全性高，有研究证实ENB技术可安全应用于高风险患者如合并重度慢性阻塞性肺病（chronic obstructive pulmonary disease, COPD）。Towe等^[18]^对341例患者行ENB检查，其中COPD占比26%，总体气胸发生率为7.5%，需留置胸管率为3.1%，多因素分析并发症的危险因素与肺功能无关。Towe等^[19]^对NAVIGATE研究分析，纳入448例COPD患者和541例非COPD患者，两者总体并发症相似（7.4%*vs*7.8%, *P*=0.90），气胸发生率低于非COPD患者（2.7%*vs*3.7%, *P*=0.47），多因素分析COPD并不是并发症的危险因素。

### 3.2 外科手术前或放射治疗前定位

难以触及的PPL的术中定位是胸外科的难题，目前常采用的方法是CT引导下经皮Hook-wire定位，但伴有定位导丝移位、出血等风险，ENB的出现提供了新的定位方法，靠近病灶或在脏层胸膜注射染料、硬化剂等标记病灶位置，提高外科手术的安全性和准确性。Luo等^[20]^报道经ENB引导至病灶附近注射混合亚甲蓝的纤维蛋白胶可辅助定位无法触及的PPL，术中可明显触及病灶，提高定位准确性，染料并不影响病理诊断。但部分长期抽烟患者的脏层胸膜染色不易发现，因此Hachey等^[21]^通过ENB注入吲哚菁绿荧光染色剂，在荧光胸腔镜下更直观切除。此外，通过ENB定位可明显缩短患者的手术等待时间，Bolton等^[22]^比较了微创肺部手术前的ENB引导下定位与CT引导下定位，发现两种方法定位耗时相似，但ENB定位后至手术的等待时间明显短于CT定位（26 min*vs*189 min），分析原因为CT组术前定位地点与手术地点不在同一房间，需要转移患者和长时间等待。ENB的这一优势可与微创胸外科优化融合，仅通过一次麻醉即可完成肺部病灶的“诊断、定位、手术”一体化诊疗模式，满足肺癌早发现早诊断早治疗的临床需求。

在影像引导放射治疗中，基准标记物作为肿瘤位置的可见替代物，既往经胸放置伴有较高气胸率并发症，而ENB可安全引导放置基准标记物，提高放疗准确性。Anantham等^[23]^2007年报道一项ENB引导下立体定向放射治疗肺肿瘤的基准定位器放置的可行性研究，总共有39个基准标记物被成功放置至8例患者体内，放置7天-10天后仍有90%的基准标记物在位可继续用于放射治疗。基于前述研究的线性基准标记物存在约10%的移位率，Schroeder等^[24]^在ENB引导下在49例患者56处肿瘤放置螺旋弹簧圈基准标记物217枚，几乎无移位，仅发生3例气胸并发症。Nabavizadeh等^[25]^同样报道经ENB引导放置弹簧圈作基准标记物，气胸发生率低，基准位置可靠，移位率低，放疗全程基准位移小于7 mm、5 mm和2 mm分别是98%、96%和67%。Mark等^[26]^分析了NAVIGATE研究中ENB引导放置基准标记物的258例患者亚组分析，每个患者平均放置2.2±1.7 FMs（中位数为1.0 FMs），99.2%的患者准确定位，随访影像显示94.1%（239/254）的标记物仍在原位，操作相关的气胸发生率位5.4%（14/258），2级以上的不良事件率为3.1%（8/258），与操作相关的4级以上呼吸衰竭发生率为1.6%（4/258），无出血事件发生。

### 3.3 治疗

ENB的临床应用是早期肺癌微创诊断的重大突破，也为肺癌患者的局部治疗提供了可靠路径。对于一些不能外科手术的患者，如合并严重心肺功能问题而不能耐受手术患者、不愿意手术或者复发转移以后不愿再次手术但又新发PPL的患者，可利用ENB引导经支气管到达或接近病灶进行近距离放疗、射频消融、微波消融、光动力等局部治疗。但ENB引导经支气管的局部治疗的有效性、安全性、远期疗效及最佳适应症等仍需更多的前瞻性临床研究和长期随访确认。

ENB引导经支气管放射治疗：2006年Harms等^[27]^成功应用ENB引导支气管内近距离放疗无法手术治疗的18例周围型肺癌患者，通过ENB寻找病灶，然后用支气管内超声确认病变位置，并沿活检通道插入放疗导管，结果表明50%患者肿瘤疗效达到完全缓解，未出现严重并发症。

ENB引导经支气管射频消融治疗：射频消融是通过使用电磁波与射频交流电对肿瘤细胞起热效应消融的治疗方法，消融部位病理组织学提示肺泡结构破坏并凝固性坏死。Tsushima等^[28]^探索经支气管对绵羊肺进行射频消融治疗的可行性。Santo等^[29]^报道19例患者进行ENB引导下射频消融治疗肺癌，可减少皮肤穿刺和探针调整次数，可有效辅助射频消融。Xie等^[30]^应用于3例患者证实ENB引导经支气管射频消融对不能手术的Ia期肺癌或转移瘤患者安全有效。

ENB引导经支气管微波消融治疗：随着ENB技术的成熟，匹配ENB的经支气管微波消融技术也逐渐成熟：柔软的微波消融针，更能适应肺部错综复杂的支气管结构；结合水循环冷却技术，使输出功率达到足以灭活一定范围内的肿瘤细胞，即可局部治疗甚至治愈肺癌。目前文献对于经支气管微波消融治疗PLL的报道则较少。上海胸科医院2016年5月成功施行国内首例ENB精确引导下经支气管微波消融术。

ENB引导经支气管光动力治疗：光动力治疗是利用光动力效应进行疾病诊断和治疗的一种新技术，其光动力效应是一种有氧分子参与的伴随生物效应的光敏化反应，通过特定波长的激光照射使组织吸收的光敏剂受到激发，而激发态的光敏剂又把能量传递给周围的氧，生成活性很强的单态氧，单态氧和相邻的生物大分子发生氧化反应，产生细胞毒性作用，进而导致细胞受损乃至死亡。Ke-Cheng Chen等^[31]^通过ENB引导对3例肺结节患者进行光动力治疗，肺结节平均直径为21.3 mm，导航平均时间为14.3 min，无严重并发症发生，1例患者术后1个月出现皮肤过敏，随访CT显示所有患者均有明显的肿瘤缩小。

## 4 展望

自20世纪90年代中期ENB问世以来，对适宜开展的患者和病变、以及影响ENB性能的技术因素的研究已经取得了长足的进展。随着ENB临床应用广泛开展，不断证实其安全有效，同时ENB经自然腔道微创、无辐射伤害等优点，与其他技术优化融合，仅通过一次麻醉即可完成PPL的“诊断、定位、手术/局部治疗”一体化诊疗模式，满足肺癌早发现早诊断早治疗的临床需求，未来有可能改变肺癌的诊断和治疗方式。目前美国胸科医师学会指南对于传统支气管镜难以触及的PPL，推荐有相关设备和经验的中心可使用ENB引导活检，推荐等级为1C^[32]^。最新美国国立综合癌症网络（National Comprehensive Cancer Network, NCCN）指南亦指出肺外1/3处PPL推荐使用ENB和径向EBUS等尽可能微创和高准确率的诊断方法^[33]^。《中国肺小结节术前辅助定位技术专家共识（2019版）》指出ENB辅助定位可以有效提高手术的安全性及结节切除的成功率^[34]^。

但ENB仍存在一定的局限性，对PPL的诊断率仍不能令人满意，定位的精准度受到支气管镜技术的制约，定位操作步骤相对繁琐，此外检查成本相对昂贵，限制了其在临床上广泛普及。ENB作为“地图”的虚拟气道重建来自操作前采集的CT数据，然而呼吸运动可能会改变病变位置。此外，气管镜置入靶段气管也可能引起显著的病变移位。由于病变的实际位置可能由于多种原因而改变，导致活检错误，因此需要实时确认PPL的位置或实时跟踪，目前已有锥形束CT被应用于临床三维确认位置。此外，如术前胸膜标记定位的最佳染料、注入染料的最佳剂量以及最合适的靶标记点等问题仍需进一步探索。

展望未来，全程引导、PPL位置的实时确认和可视化、实时跟踪活检工具、开发更先进的治疗工具和成像技术等仍是未来ENB临床应用需要解决的难题，从而使ENB引导下的诊断和治疗更精准、更有效。
